# Adipogenic Gene Expression in Gilthead Sea Bream Mesenchymal Stem Cells from Different Origin

**DOI:** 10.3389/fendo.2016.00113

**Published:** 2016-08-22

**Authors:** Cristina Salmerón, Natàlia Riera-Heredia, Joaquim Gutiérrez, Isabel Navarro, Encarnación Capilla

**Affiliations:** ^1^Department of Cell Biology, Physiology and Immunology, Faculty of Biology, University of Barcelona, Barcelona, Spain

**Keywords:** MSCs, adipogenesis, adipocyte, bone, *Sparus aurata*

## Abstract

During the last decades, adipogenesis has become an emerging field of study in aquaculture due to the relevance of the adipose tissue in many physiological processes and its connection with the endocrine system. In this sense, recent studies have translated into the establishment of preadipocyte culture models from several fish species, sometimes lacking information on the mRNA levels of adipogenic genes. Thus, the aim of this study was to determine the gene expression profile of gilthead sea bream (*Sparus aurata*) primary cultured mesenchymal stem cells (MSCs) from different origin (adipose tissue and vertebra bone) during adipogenesis. Both cell types differentiated into adipocyte-like cells, accumulating lipids inside their cytoplasm. Adipocyte differentiation of MSCs from adipose tissue resulted in downregulation of several adipocyte-related genes (such as *lpl, hsl, ppar*α, *ppar*γ and *gapdh2*) at day 4, *gapdh1* at day 8, and *fas* and *ppar*β at day 12. In contrast, differences in *lxr*α mRNA expression were not observed, while *g6pdh* levels increased during adipocyte maturation. Gapdh and Pparγ protein levels were also detected in preadipocyte cultures; however, only the former increased its expression during adipogenesis. Moreover, differentiation of bone-derived cells into adipocytes also resulted in the downregulation of several adipocyte gene markers, such as *fas* and *g6pdh* at day 10 and *hsl, ppar*β, and *lxr*α at day 15. On the other hand, the osteogenic genes *fib1a, mgp*, and *op* remained stable, but an increase in *runx2* expression at day 20 was observed. In summary, the present study demonstrates that gilthead sea bream MSCs, from both adipose tissue and bone, differentiate into adipocyte-like cells, although revealed some kind of species- and cell lineage-specific regulation with regards to gene expression. Present data also provide novel insights into some of the potential key genes controlling adipogenesis in gilthead sea bream that can help to better understand the regulation of lipid storage in fish.

## Introduction

Traditionally adipose tissue was considered a mere energy store, synthesizing and accumulating triglycerides during caloric excess periods and releasing fatty acids and glycerol when needed, as under nutritional restriction. However, this changed with the discovery of the adipose tissue-produced hormone leptin in 1994 (1). Adipocytes and cells in the stromal vascular fraction of adipose tissue produce many hormones, cytokines, and other molecules, with more than 50 described to date also in fish (2–8), which act on the central nervous system and peripheral organs regulating several processes, such as glucose and lipid metabolism (9, 10). Thus, it is now recognized that adipose tissue is an active contributor to the regulation of whole-body energy homeostasis.

Adipose tissue grows by increasing the size of existing adipocytes (hypertrophy), its number based on the formation of new adipocytes from precursor cells (hyperplasia), or both (11). These two adipocyte developmental types occur not only during the early life stages but also throughout life (12, 13). In addition to adipocytes, the adipose tissue also comprises a stromal vascular fraction formed by a heterogeneous population of cells, containing mesenchymal stem cells (MSCs) that include multipotent cells with the ability to differentiate into adipocytes, chondrocytes, and osteoblasts, among other cell lineages (14, 15). The process of adipocyte differentiation is divided in two steps and is influenced by hormones, growth factors, cytokines, and nutrients. First, the multipotent MSCs undergo a process known as determination (16, 17). This process results in cells that are morphologically similar to fibroblasts, which appear identical to MSCs, but are only able to differentiate into adipocytes. As a result, the cells in this post-determination state are called preadipocytes or adipoblasts. The second stage, the proper differentiation, consists in the formation of structurally mature adipocytes from preadipocytes and is commonly known as adipogenesis, where changes in cellular morphology, hormone sensitivity, and secretory capacity of the cells occur (18, 19). These changes are regulated through the coordinated expression of mainly transcription factors, which in turn act to activate transcription of genes that produce the adipocyte phenotype (20, 21). The peroxisome proliferator-activated receptor γ (Pparγ) is the central regulator of adipogenesis and is responsible for activating a number of genes involved in fatty acid binding, uptake, and storage, including lipoprotein lipase (*lpl*) or phosphoenolpyruvate carboxykinase, among others.

The interest in the adipogenic process and its regulation in fish has increased in the last years, because in aquaculture, the excessive fat accumulation experienced by some cultured species is generally perceived as an undesirable trait by the consumers and also has negative effects in terms of production, product lifetime, and fish health. Therefore, several primary cultures of preadipocytes have been established to better understand adipogenesis and its endocrine regulation in fish, including Atlantic salmon (*Salmo salar*) (8), red sea bream (*Pagrus major*) (22), rainbow trout (*Oncorhynchus mykiss*) (23), large yellow croaker (*Pseudosciaena crocea*) (24), grass carp (*Ctenopharyngodon idella*) (25), gilthead sea bream (*Sparus aurata*) (26), and common carp (*Cyprinus carpio*) (27). However, knowledge on the gene expression pattern during fish adipogenesis is usually scarce, especially in sparids, with only two microarray studies reported to date in salmonids, one in Atlantic salmon (7) and the other in rainbow trout (28).

Adipocytes and osteoblasts arise from a common precursor cell, which after the induction of certain transcription factors, differentiates into each one of these two cell types. As mentioned before, Pparγ is the master transcription factor for adipocyte differentiation, while runt-related transcription factor 2 (Runx2) is considered the one regulating osteogenic differentiation (20). The prevalence of skeletal malformations in hatchery-reared fish stimulated the establishment of cell culture models to study the mechanisms of bone formation and development in fish. The osteoblast models described to date have been two cell lines derived from vertebra and branchial arch of gilthead sea bream (29), one cell line derived from zebrafish (*Danio rerio*) calcified tissues (30), and three primary cultures; one derived from gilthead sea bream vertebra bone (31) and two of Atlantic salmon, one from white muscle precursor cells (32) and one from visceral fat precursor cells (33). Using the bone-derived cells from vertebra of gilthead sea bream, we have demonstrated that they are multipotent stem cells as they can be differentiated into either osteoblasts or adipocyte-like cells using an osteogenic or an adipogenic differentiation medium (DM), respectively (31). Nevertheless, the changes that occur in the transcriptional profile of these cells during such processes have not yet been investigated either.

Furthermore, another interest of the study of these cell culture models is that during the last decade in mammals, it has been speculated that the infiltration of bone marrow adipocytes during the development of osteoporosis in the elderly can be due to osteo-adipogenic transdifferentiation (34). In fish, the hematopoietic organ is the head kidney, and the bone marrow is from the beginning of its development filled with adipocytes, besides nerves, blood vessels, and connective tissue cells (35); therefore, we can hypothesize that such a process of adipocyte transdifferentiation from cells of the osteoblastic lineage may be also occurring in the adult, contributing to the whole fat content of the fish. As a first step toward exploring that possibility and using an *in vitro* approach, the aims of the current study were to (1) determine the transcriptional profile of gilthead sea bream preadipocytes during adipogenesis and (2) compare it with the gene expression pattern observed throughout the differentiation of bone-derived MSCs into adipocyte-like cells. To this end, we used primary cell cultures of gilthead sea bream precursor cells obtained from visceral adipose tissue and vertebra bone and analyzed well-characterized adipocyte differentiation markers and some osteogenic markers at different adipogenic stages.

## Materials and Methods

### Animal Care

Animal care and experimental procedures complied with the Guidelines of the European Union Council (86/609/EU) and were approved by the Ethics and Animal Care Committee of the University of Barcelona, following the Catalan government-established norms and procedures (permit numbers DAAM 7951; CEEA 169/14, and DAAM 6759; CEEA 243/12 for the preparation of primary cultures derived from adipose tissue or bone, respectively).

### Fish

Gilthead sea bream were obtained from a fish farm in Northern Spain and maintained in the animal facilities of the Faculty of Biology at the University of Barcelona. Fish were kept in 200 L fiberglass tanks under 12-h light/12-h dark photoperiod at 21 ± 1°C, pH 7.5–8, 31–38% salinity and >80% oxygen saturation and fed *ad libitum* twice daily with a commercial diet (Skretting España SA, Burgos, Spain).

### Preadipocytes and Bone Cells Cultures

All plasticware for tissue culture was obtained from Nunc (Barcelona, Spain); and all the reagents were purchased from Sigma–Aldrich (Tres Cantos, Spain), unless stated otherwise. Cells were incubated at 23°C with 2.5% CO_2_ during the whole duration of the experiments.

#### Preadipocytes Cultures

Three or four juvenile gilthead sea bream of an average weight of 104 g were used for each culture. Preadipocytes were collected by mechanical disruption and enzymatic digestion of the visceral adipose tissue as described previously (26), plated at a density of 4.3 × 10^4^ cells/cm^2^ in gelatin-pretreated 6-well plates and maintained in growth media (GM) composed of Dulbecco’s Modified Eagle Medium (DMEM) with 10% fetal bovine serum (FBS), 1% antibiotic/antimycotic solution (A/A), and supplemented with 60 mM NaCl. Cells were continuously cultured in GM until day 8, once confluence was reached, and then changed to a DM containing GM plus 10 μg/mL porcine insulin, 0.5 mM 1-methyl-3-isobutylxanthine (IBMX), 0.25 μM dexamethasone, and 5 μL/mL lipid mixture (including cholesterol and fatty acids from cod liver oil) to induce adipocyte differentiation. After 3 days, the culture conditions were changed to an adipocyte medium (AM), consisting of GM plus lipid mixture (5 μL/mL), to keep the cells already differentiating until the end of the culture. Preadipocytes samples were collected at days 4 and 8 of culture (days −4 and 0, respectively) and samples of adipocytes at days 4, 8, and 12 after induction of differentiation. Prior to harvesting, cells were washed once with phosphate-buffered saline (PBS), recovered with TRI Reagent (Ambion, Alcobendas, Spain) using a cell scraper, then transferred to an RNase-free polypropylene tube, and kept at −80°C until RNA and protein extraction.

#### Bone Cultures

A total of six juvenile gilthead sea bream of an average weight of 30 g were used for each culture. Bone-derived cells were isolated by mechanical disruption and enzymatic digestion of vertebra bone as described previously (31). Cells and small vertebra fragments were plated with GM supplemented with 19 mM NaCl and 1% fungizone (Invitrogen Life Technologies, Alcobendas, Spain), in a 10 cm culture dish. After 1 week, the fragments were removed and the attached cells collected with 0.25% trypsin–EDTA (Invitrogen Life Technologies, Alcobendas, Spain) and plated into new 10 cm plates with fresh GM. From here, the cells were routinely subcultured every time the cells reached about 70–80% confluence and used for a maximum of 10 passages. Differentiation into adipocyte-like cells was achieved as previously described (31). Briefly, 70–80% confluent cells were trypsinized from 10 cm culture dishes, seeded (1 × 10^4^ cells/cm^2^), and cultured in 6-well plates with GM. The following day (i.e., day 0), media was changed first to DM, and 3 days later, changed to AM using the same experimental settings as explained in Section “Preadipocytes Cultures.” Cell samples for RNA extraction were obtained at days 5, 10, 15, and 20 as described in Section “Preadipocytes Cultures.”

### RNA and Protein Extraction

Simultaneous extraction of RNA and proteins from a single cell sample was performed using TRI Reagent (Ambion, Alcobendas, Spain) and following the manufacturer’s recommendations.

#### RNA Extraction and cDNA Synthesis

Total RNA was dissolved in DEPC-treated water (RNase-free) and stored at −80°C. RNA was quantified using a NanoDrop 2000 spectrophotometer (Thermo Scientific, Alcobendas, Spain), and its integrity was analyzed by 1% (m/v) agarose gel electrophoresis. To eliminate any residual genomic DNA, total RNA (250 ng from preadipocytes and 1 μg from bone cells) was treated with DNase I (Invitrogen, Alcobendas, Spain) and converted into cDNA using the Transcriptor First Strand cDNA Synthesis Kit (Roche, Sant Cugat del Valles, Spain), following the manufacturer’s recommendations.

#### Protein Extraction

Protein was extracted from the preadipocytes samples only and dissolved in RIPA buffer (Tris-HCl 50 mM, pH 7.4, NaCl 150 mM, EDTA 1 mM, NP-40 1%, Na-deoxycholate 0.25%, PMSF 1 mM, Na_3_VO_4_ 1 mM, NaF 1 mM, and protease inhibitor cocktail). Then, samples were homogenized using a “pellet pestle” for microtubes on ice, mixed in an orbital during 1 h at 4°C, and the supernatant was recovered after centrifugation during 30 min at maximum speed at 4°C. Total protein concentration was determined (595 nm) by Bradford assay using bovine serum albumin (BSA) as the standard protein (36).

### Quantitative PCR Analysis

In order to characterize the transcriptional profile occurring during adipocyte differentiation in gilthead sea bream, important genes implicated in adipogenesis and energy metabolism regulation were analyzed by quantitative PCR (qPCR). The genes comprise the following enzymes: fatty acid syntase (*fas*), *lpl*, glucose-6-phosphate dehydrogenase (*g6pdh*), hormone-sensitive lipase (*hsl*), and glyceraldehyde 3-phosphate dehydrogenase (*gapdh*) 1 and 2; and the following transcription factors: *ppar*α, *ppar*β, *ppar*γ, and liver X receptor alpha (*lxr*α). Moreover, the expression of four osteogenic genes, fibronectin 1a (*fib1a*), matrix Gla protein (*mgp*), osteopontin (*op*), and *runx2*, was determined in the cells derived from bone. In addition, elongation factor 1α (*ef1*α), ribosomal protein S18 (*rps18*), and 18S ribosomal RNA (*18s*) were tested as reference genes. qPCR was performed using a CFX384 thermocycler (Bio-Rad, El Prat de Llobregat, Spain) as previously described (37). Each qPCR reaction was performed in triplicate in a total volume of 5 μL, containing 2.5 μL of the iTaq Universal SYBR Green supermix (Bio-Rad, El Prat de Llobregat, Spain), 0.125–1.25 ng of cDNA template, 250–500 nM of each primer (Table 1), and milliQ water. Samples were amplified as follows: 95°C for 3 min, and then 40 cycles of 95°C for 10 s, followed by annealing 57–69°C for 30 s (primer-dependent, Table 1), followed by dissociation step from 55 to 95°C with a 0.5°C increase every 5 s. A standard curve dilution series of a cDNA sample pool was constructed to determine the qPCR efficiency of each primer pair (Table 1), which was calculated using the CFX Manager Software (Bio-Rad). No template control (NTC), no reverse transcription control (RTC), and PCR control (PCR) were used to determine the overall performance of each qPCR assay. Relative expression levels of the target genes were determined by the Pfaffl method (38) using correction for primer efficiencies and normalizing the quantification cycle (Cq) value of each gene registered during the annealing step to that of *rps18* and *ef1*α, the most stable reference genes among the different culture stages (*P* > 0.05) using the CFX Manager Software (Bio-Rad). Data from preadipocytes and bone cells were obtained from 4–5 and 5–6 independent cultures, respectively.

**Table 1:** **Primers used for real-time quantitative PCR**.

Gene	Primer sequence (5′→3′)	Tm (°C)	Product size (bp)	*E* (%)	Acc. Num.
*lpl*_FW	GAGCACGCAGACAACCAGAA	60	135	98.1	AY495672
*lpl*_RV	GGGGTAGATGTCGATGTCGC				
*fas*_FW	TGGCAGCATACACACAGACC	60	78	102.0	AM952430
*fas*_RV	CACACAGGGCTTCAGTTTCA				
*g6pdh*_FW	CAGAATGAAAGATGGGATGGAGTC	60	176	102.9	AY754640
*g6pdh*_RV	TTCAGGTAAATGGCTTCGTTCG				
*hsl*_FW	GCTTTGCTTCAGTTTACCACCATTTC	60	122	99.6	EU254478
*hsl*_RV	GATGTAGCGACCCTTCTGGATGATGTG				
*gapdh1*_FW	CCAGCCAGAACATCATCC	60	190	103.5	DQ641630
*gapdh1*_RV	GCAGCCTTGACGACCTTC				
*gapdh2*_FW	CATGAAGCCAGCAGAGATCC	57	196	105.5	FM145063
*gapdh2*_RV	GGTGGCCGGGTCATATTTC				
*ppar*α_FW	TCTCTTCAGCCCACCATCCC	62	116	104.2	AY590299
*ppar*α_RV	ATCCCAGCGTGTCGTCTCC				
*ppar*β_FW	AGGCGAGGGAGAGTGAGGATGAGGAG	69	188	108.3	AY590301
*ppar*β_RV	CTGTTCTGAAAGCGAGGGTGACGATGTTTG				
pparγ_FW	CGCCGTGGACCTGTCAGAGC	66	171	96.0	AY590304
pparγ_RV	GGAATGGATGGAGGAGGAGGAGATGG				
lxrα_FW	GCACTTCGCCTCCAGGACAAG	62	107	88.0	FJ502320
lxrα_RV	CAGTCTTCACACAGCCACATCAGG				
*fib1a*_FW	CGGTAATAACTACAGAATCGGTGAG	60	104	101.8	FG262933
*fib1a*_RV	CGCATTTGAACTCGCCCTTG				
*mgp*_FW	TGTGTAATTTATGTAGTTGTTCTGTGGCATCTCC	68	244	81.8	AY065652
*mgp*_RV	CGGGCGGATAGTGTGAAAAATGGTTAGTG				
*op*_FW	AAAACCCAGGAGATAAACTCAAGACAACCCA	68	153	85.0	AY651247
*op*_RV	AGAACCGTGGCAAAGAGCAGAACGAA				
*runx2*_FW	ACCCGTCCTACCTGAGTCC	60	122	96.1	JX232063
*runx2*_RV	AGAAGAACCTGGCAATCGTC				
*ef1*α_FW	CTTCAACGCTCAGGTCATCAT	60	263	85.0	AF184170
*ef1*α_RV	GCACAGCGAAACGACCAAGGGGA				
*rps18*_FW	AGGGTGTTGGCAGACGTTAC	60	164	98.3	AM490061
*rps18*_RV	CTTCTGCCTGTTGAGGAACC				
*18s*_FW	TGACGGAAGGGCACCACCAG	60	158	91.0	AY550956
*18s*_RV	AATCGCTCCACCAACTAAGAACGG				

*lpl, lipoprotein lipase; fas, fatty acid synthase; g6pdh, glucose-6-phosphate dehydrogenase; hsl, hormone-sensitive lipase; gapdh1, glyceraldehyde 3-phosphate dehydrogenase 1; gapdh2, glyceraldehyde 3-phosphate dehydrogenase 2; pparα, peroxisome proliferator-activated receptor alpha; pparβ, peroxisome proliferator-activated receptor beta; pparγ, peroxisome proliferator-activated receptor gamma; lxrα, liver X receptor alpha; fib1a, fibronectin 1a; mgp, matrix Gla protein; op, osteopontin; runx2, runt-related transcription factor 2; ef1α, elongation factor 1 alpha; rps18, ribosomal protein S18; 18s, 18S ribosomal RNA; FW, forward primer; RV, reverse primer; Tm, annealing temperature; bp, base pair; E, amplification efficiency; Acc. Num., accession number*.

### Western Blotting

For Western blot analyses, 8 μg of protein were loaded in each lane. Proteins were separated by sodium dodecyl sulfate polyacrylamide gel electrophoresis (SDS-PAGE) (12%) and transferred to polyvinylidene difluoride (PVDF) membranes (Bio-Rad, El Prat de Llobregat, Spain) overnight at 4°C and 100 mA. Reversible Ponceau staining was used as a loading control (39). The PVDF membranes were blocked with 5% skimmed milk powder in Tris-Buffered Saline and Tween 20 (TBS-T) for 1 h 15 min and probed with rabbit polyclonal Pparγ (sc-7196) and goat polyclonal Gapdh (sc-20357) primary antibodies (Santa Cruz Biotechnology, CA, USA) at a dilution of 1:200 overnight at 4°C on a tube rotator. Membranes were washed in TBS-T and probed with a horseradish peroxidase-conjugated anti-rabbit (sc-2004) or anti-goat (sc-2020) secondary antibody (Santa Cruz Biotechnology, CA, USA) at a dilution of 1:10,000 for 1 h 15 min. Membranes were washed with TBS-T and chemiluminescent detection performed using an enhanced chemiluminescence kit (Pierce ECL Western blotting Substrate; Thermo Scientific, Alcobendas, Spain). Western blotting results were obtained from two independent cultures, and band intensities were quantified by scanning densitometry using ImageJ software (National Institutes of Health, Bethesda, MD, USA) and normalized to Ponceau staining.

### Oil Red O Staining

To evaluate adipocyte differentiation in the bone-derived cells, intracellular neutral lipid accumulation was analyzed by Oil red O staining as explained in Ref. (31). Cells were grown as explained in Section “Bone Cultures” and samples obtained at days 5 and 15 of culture development. Quantification of cell lipid content was calculated as the absorbance measured at 490 nm divided by the read at 630 nm corresponding to cell protein content, which was obtained after Comassie blue staining for 1 h and dye extraction by incubation of the cells with 85% propylene glycol during 3 h at 60°C (31). Data are presented as fold change relative to day 5 of culture.

### Statistical Analyses

Data normality and homoscedasticity were assessed using Shapiro–Wilk and Levene’s test, respectively. Independent samples’ Student’s *t*-test was used for comparison between two groups. For multiple mean comparisons of normal distributed data, one-way ANOVA was used followed by Tukey’s or Dunnett’s T3 *post hoc* tests in case of homogeneous or heterogeneous variance data, respectively. When data did not fit normal distribution, the non-parametric Kruskal–Wallis test, followed by Mann–Whitney test, were used. Statistical analyses were performed using SPSS Statistics version 20 (IBM, Armonk, NY, USA). Results were presented as mean ± SEM. *P* < 0.05 was considered to indicate a statistically significant difference. Graphs were generated using GraphPad Prism version 6.00 for Windows (GraphPad Software, La Jolla, CA, USA, www.graphpad.com).

## Results

### Gene and Protein Expression Profiles during Differentiation of Preadipocytes

**Figure 1:**
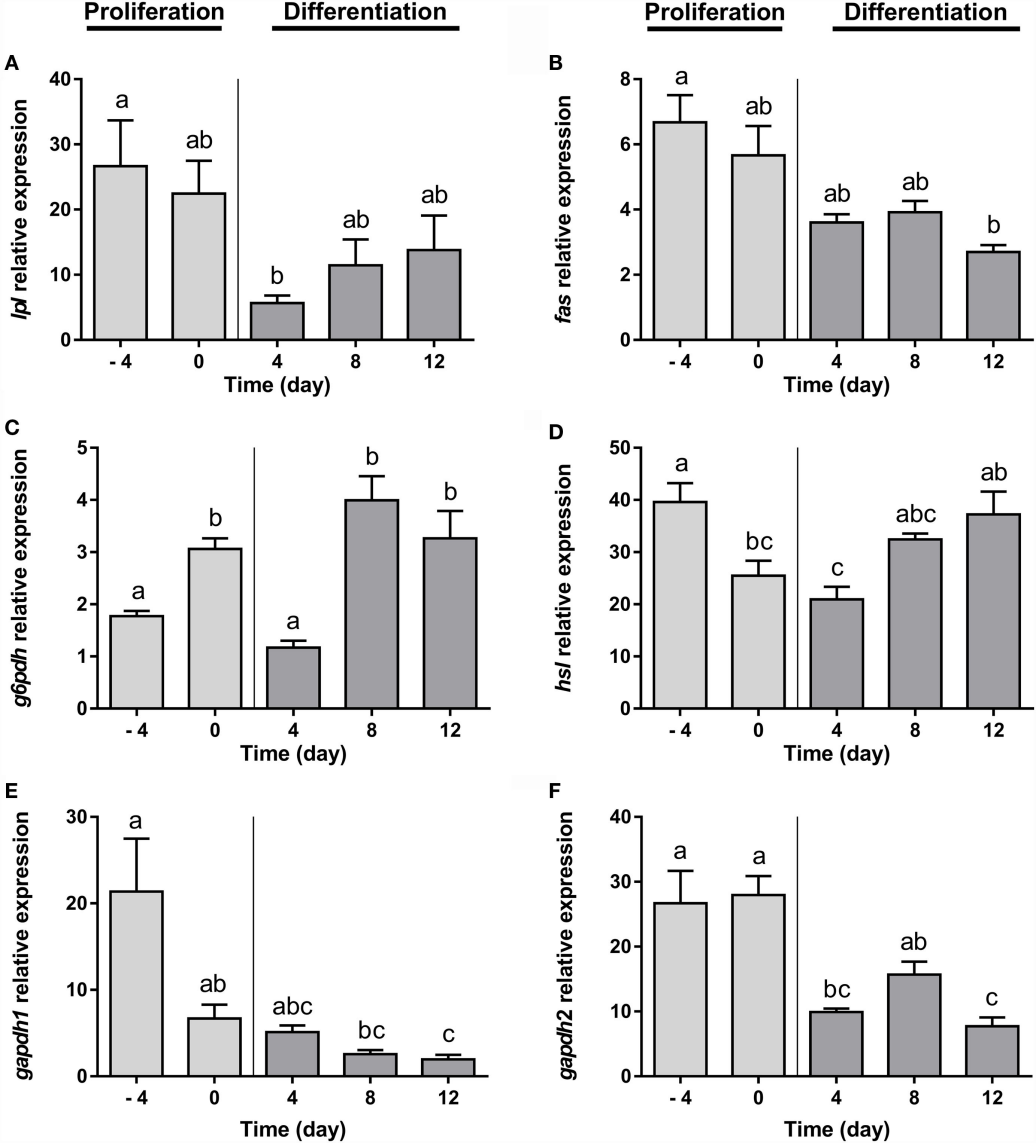
**Gene expression profile of lipid metabolism-related genes in gilthead sea bream preadipocytes during adipogenesis**. The mRNA levels of *lpl* **(A)**, *fas* **(B)**, *g6pdh* **(C)**, *hsl* **(D)**, *gapdh1* **(E)**, and *gapdh2* **(F)** were measured by quantitative real-time PCR and normalized to *ef1*α and *rps18*. Samples were taken from preadipocytes (days −4 and 0 of culture) and differentiated adipocytes (days 4, 8, and 12 of culture). Values are means ± SEM, *n* = 4–5. Bars with different letters are significantly different (*P* < 0.05). *lpl*, lipoprotein lipase; *fas*, fatty acid synthase; *g6pdh*, glucose-6-phosphate dehydrogenase; *hsl*, hormone-sensitive lipase; *gapdh1* and *2*, glyceraldehyde 3-phosphate dehydrogenase 1 and 2, respectively; *ef1*α, elongation factor 1 alpha; *rps18*, ribosomal protein S18.

Gilthead sea bream preadipocytes require the addition of a DM, a mixture of insulin, IBMX, dexamethasone, and lipid mixture, to differentiate into mature adipocytes (26). After differentiation induction, gilthead sea bream preadipocytes start to change its morphology from a fibroblast-like shape to an adipocyte-like form with an enlarged cytoplasm filled with lipids (26). The transcriptional profile during adipogenesis in gilthead sea bream preadipocytes was analyzed before (days −4 and 0) and after (days 4, 8, and 12) induction of differentiation. The mRNA levels of the early marker of adipocyte differentiation *lpl* decreased significantly from day −4 to day 4 and then gradually increased again (Figure 1A). *fas* and *g6pdh*, genes participating in the *via* of *de novo* lipogenesis from glucose and in the pentose phosphate pathway, respectively, showed opposite expression patterns, with *fas* gradually decreasing (Figure 1B), while *g6pdh* was significantly upregulated during most of the process (Figure 1C). The expression of the lipolysis-associated gene *hsl* was significantly higher in preadipocytes and late-differentiated adipocytes relative to cells at day 4 (Figure 1D). With regards to the adipocyte maturation marker *gapdh*, the expression of both isoforms (*gapdh1* and *gapdh2*) significantly diminished along with differentiation (Figures 1E,F).

**Figure 2:**
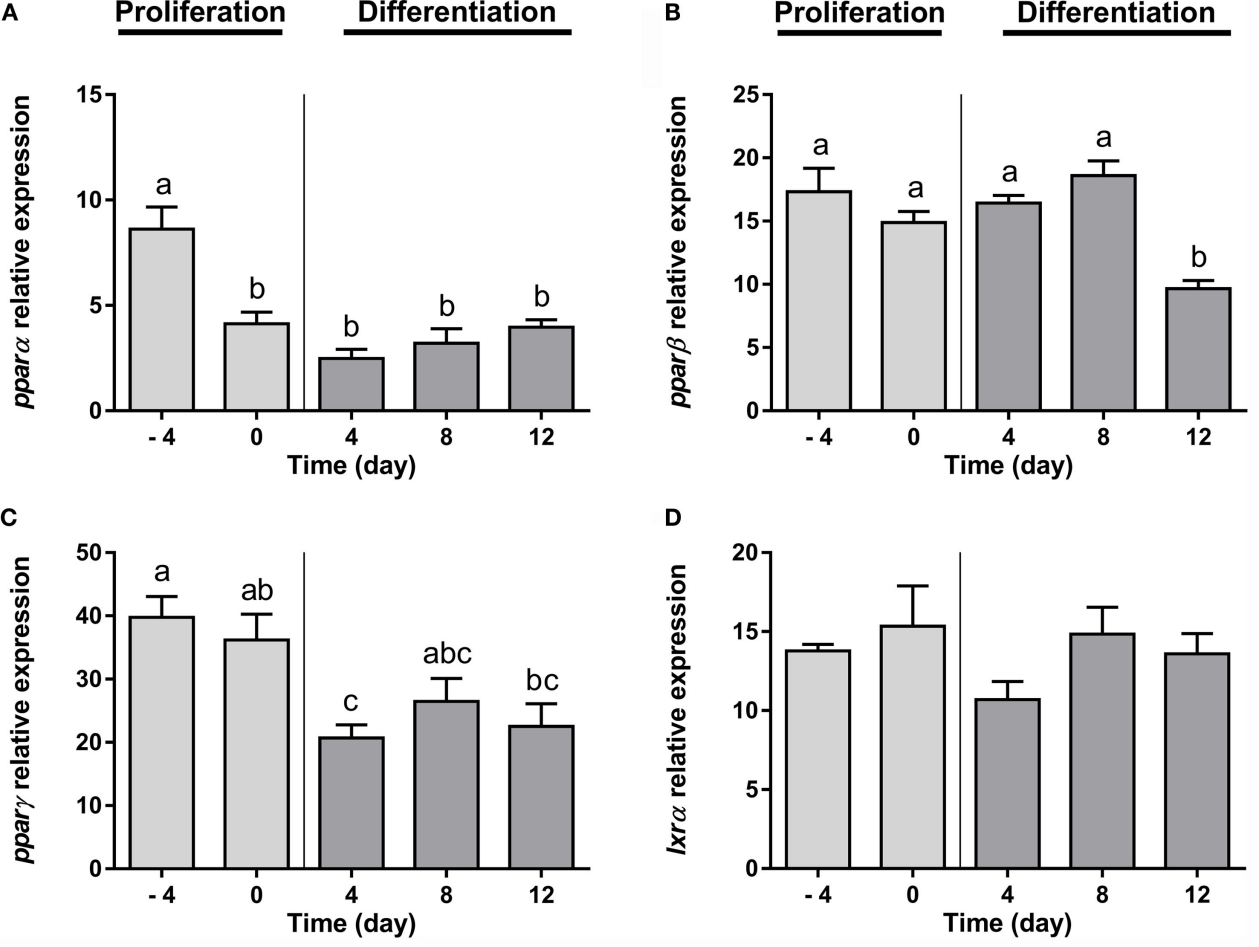
**Gene expression profile of transcription factors in gilthead sea bream preadipocytes during adipogenesis**. The mRNA levels of *ppar*α **(A)**, *ppar*β **(B)**, *ppar*γ **(C)**, and *lxr*α **(D)** were measured by quantitative real-time PCR and normalized to *ef1*α and *rps18*. Samples were taken from preadipocytes (days −4 and 0 of culture) and differentiated adipocytes (days 4, 8, and 12 of culture). Values are means ± SEM, *n* = 4–5. Bars with different letters are significantly different (*P* < 0.05). *ppar*α, β, and γ, peroxisome proliferator-activated receptor alpha, beta, and gamma, respectively; *lxr*α, liver X receptor alpha; *ef1*α, elongation factor 1 alpha; *rps18*, ribosomal protein S18.

Furthermore, regarding the expression of the transcription factors analyzed, *ppar*α and *ppar*β, nuclear receptors that regulate the beta-oxidation of fatty acids, were significantly downregulated from early and at late stages of differentiation, respectively (Figures 2A,B). The transcript levels of *ppar*γ, the nuclear receptor key in the process of adipocyte differentiation, decreased significantly after the addition of the DM (day 4), but then its expression was maintained along with adipocyte maturation (Figure 2C). Finally, the mRNA levels of *lxr*α, a nuclear receptor participating in the regulation of cholesterol homeostasis, was stable during the whole adipogenic process (Figure 2D).

**Figure 3:**
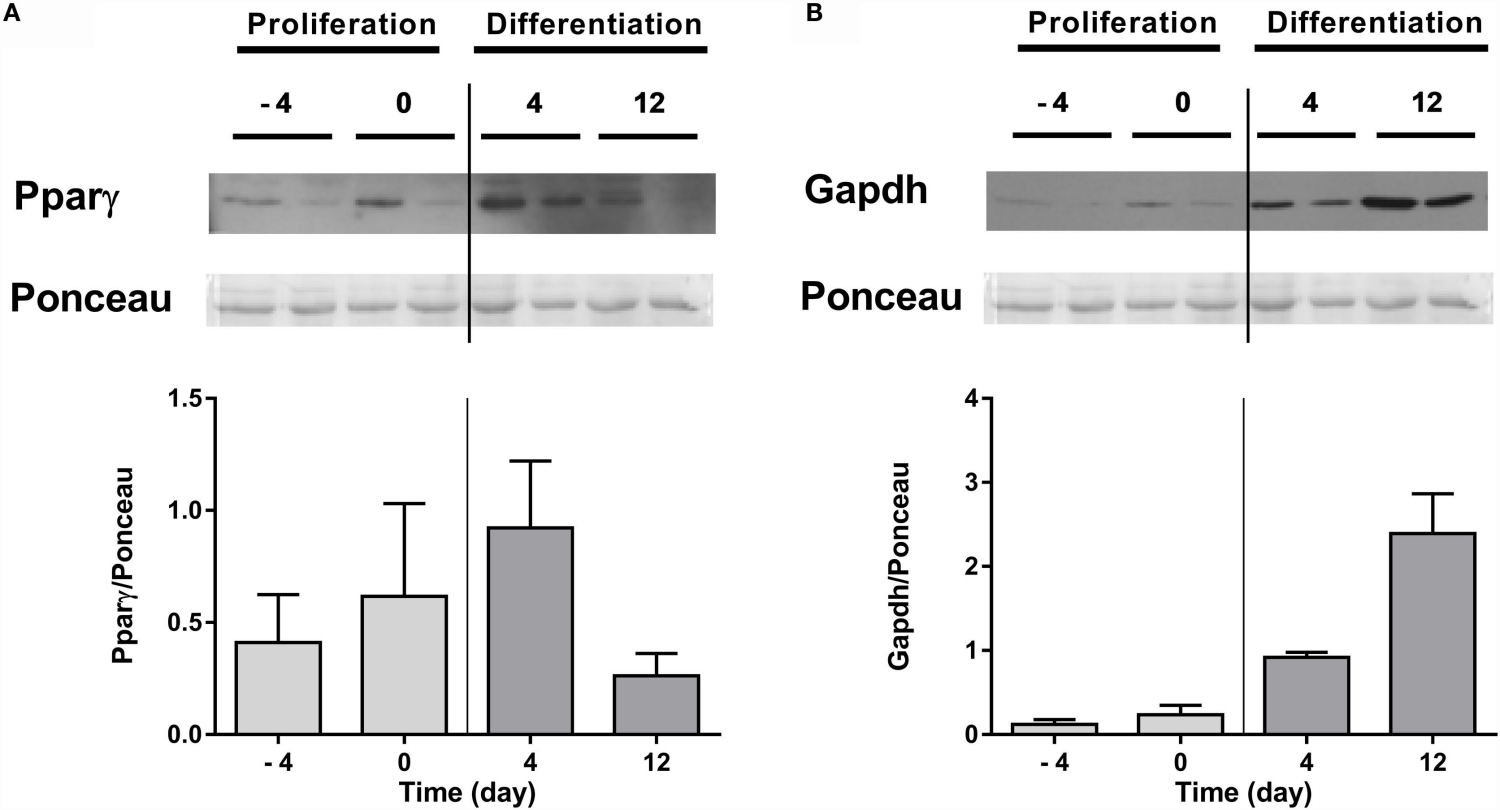
**Protein expression of Pparγ and Gapdh in gilthead sea bream preadipocytes during adipogenesis**. Representative Western blot, corresponding Ponceau, and quantification of Pparγ **(A)** and Gapdh **(B)** protein levels in preadipocytes (days −4 and 0 of culture) and differentiated adipocytes (days 4 and 12 of culture). Values are means ± SEM, *n* = 2. Pparγ, peroxisome proliferator-activated receptor gamma; Gapdh, glyceraldehyde 3-phosphate dehydrogenase.

Next, protein expression of Pparγ and Gapdh was also determined in lysates from undifferentiated (days −4 and 0) and differentiated (days 4 and 12) adipocytes. Results showed that Pparγ tended to increase up to day 4 and then diminished (Figure 3A), while Gapdh increased steadily during adipogenesis (Figure 3B).

### Lipid Accumulation during Adipogenesis of Bone-Derived Cells

**Figure 4:**
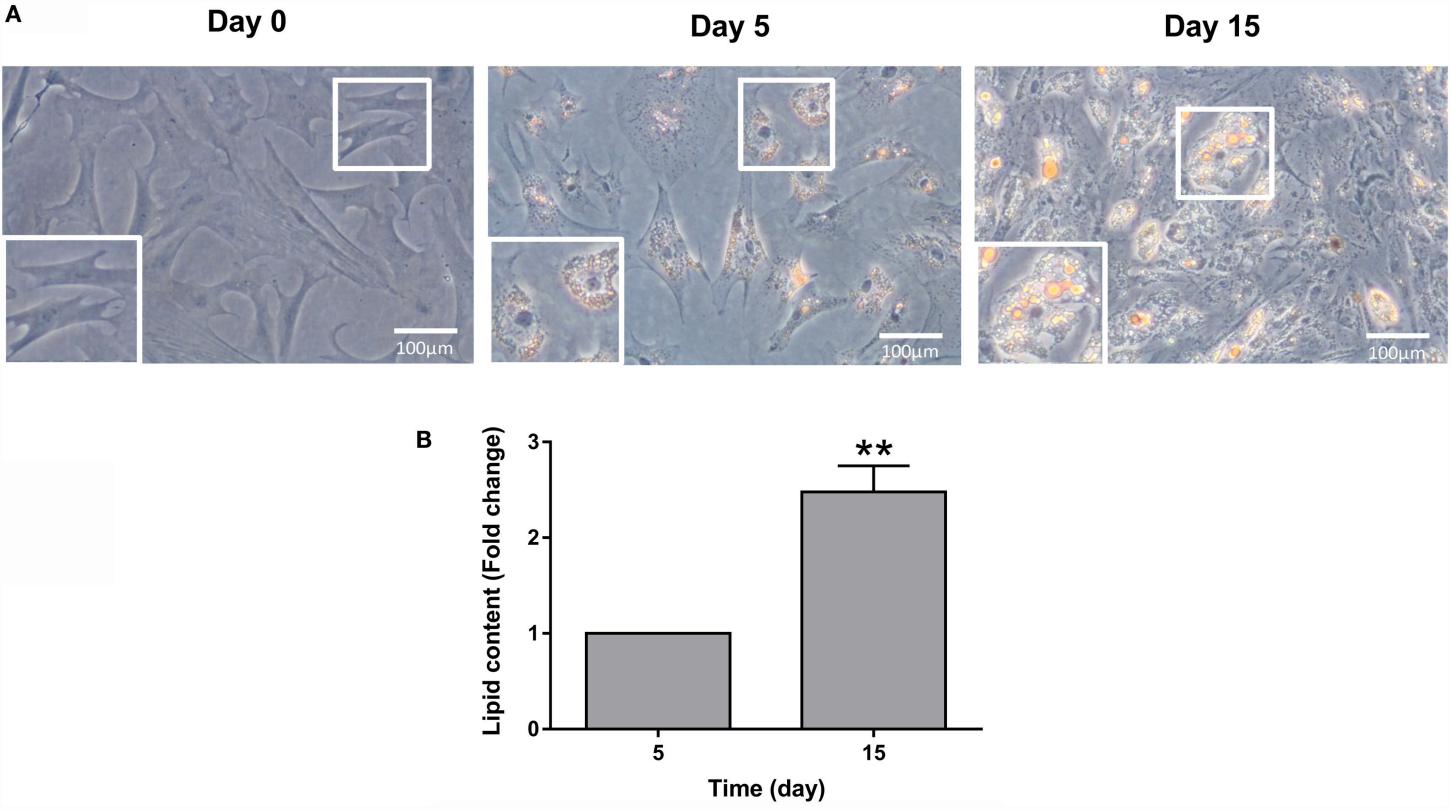
**Gilthead sea bream bone-derived cells during adipogenesis**. **(A)** Representative phase-contrast images of cells stained with Oil red O at days 0, 5, and 15 of culture. Magnification 20× and enlarged views. **(B)** Quantification of Oil red O staining at days 5 and 15 of culture. Values are mean ± SEM of five independent experiments with wells run in duplicate and presented as fold change relative to day 5 of culture (**P < 0.01).

Bone-derived MSCs of gilthead sea bream can become adipocyte-like cells using the same DM than in preadipocytes (31). Phenotypic changes from the cells with a fibroblast-like appearance to more rounded and lipid-filled cells with the morphological semblance of adipocytes were observed during adipogenesis (Figure 4A). Differentiation into adipocyte-like cells was tracked by Oil red O staining, which monitors lipid accumulation. Lipid content in the cells was gradually increasing during the process of adipocyte maturation, showing day 15 cells significant differences when compared to day 5 cells (Figure 4B).

### Gene Expression Profile during Adipogenesis of Bone-Derived Cells

**Figure 5:**
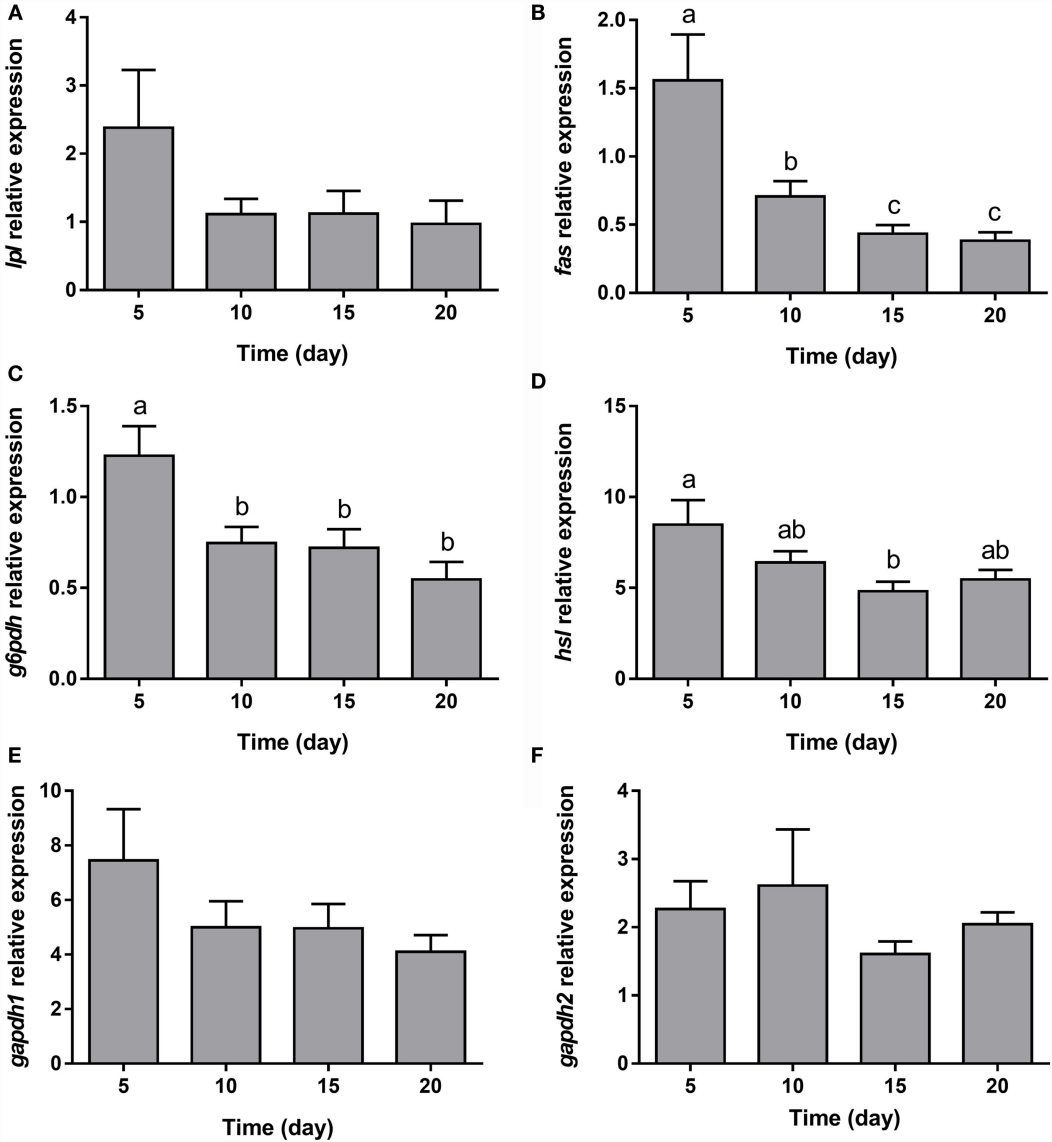
**Gene expression profile of lipid metabolism-related genes in gilthead sea bream bone-derived cells during adipogenesis**. The mRNA levels of *lpl* **(A)**, *fas* **(B)**, *g6pdh* **(C)**, *hsl* **(D)**, *gapdh1* **(E)**, and *gapdh2* **(F)** were measured by quantitative real-time PCR and normalized to *ef1*α and *rps18*. Samples were taken from adipocyte-like cells at days 5, 10, 15, and 20 of culture. Values are means ± SEM, *n* = 5–6. Bars with different letters are significantly different (*P* < 0.05). *lpl*, lipoprotein lipase; *fas*, fatty acid synthase; *g6pdh*, glucose-6-phosphate dehydrogenase; *hsl*, hormone-sensitive lipase; *gapdh1* and *2*, glyceraldehyde 3-phosphate dehydrogenase 1 and 2, respectively; *ef1*α, elongation factor 1 alpha; *rps18*, ribosomal protein S18.

**Figure 6:**
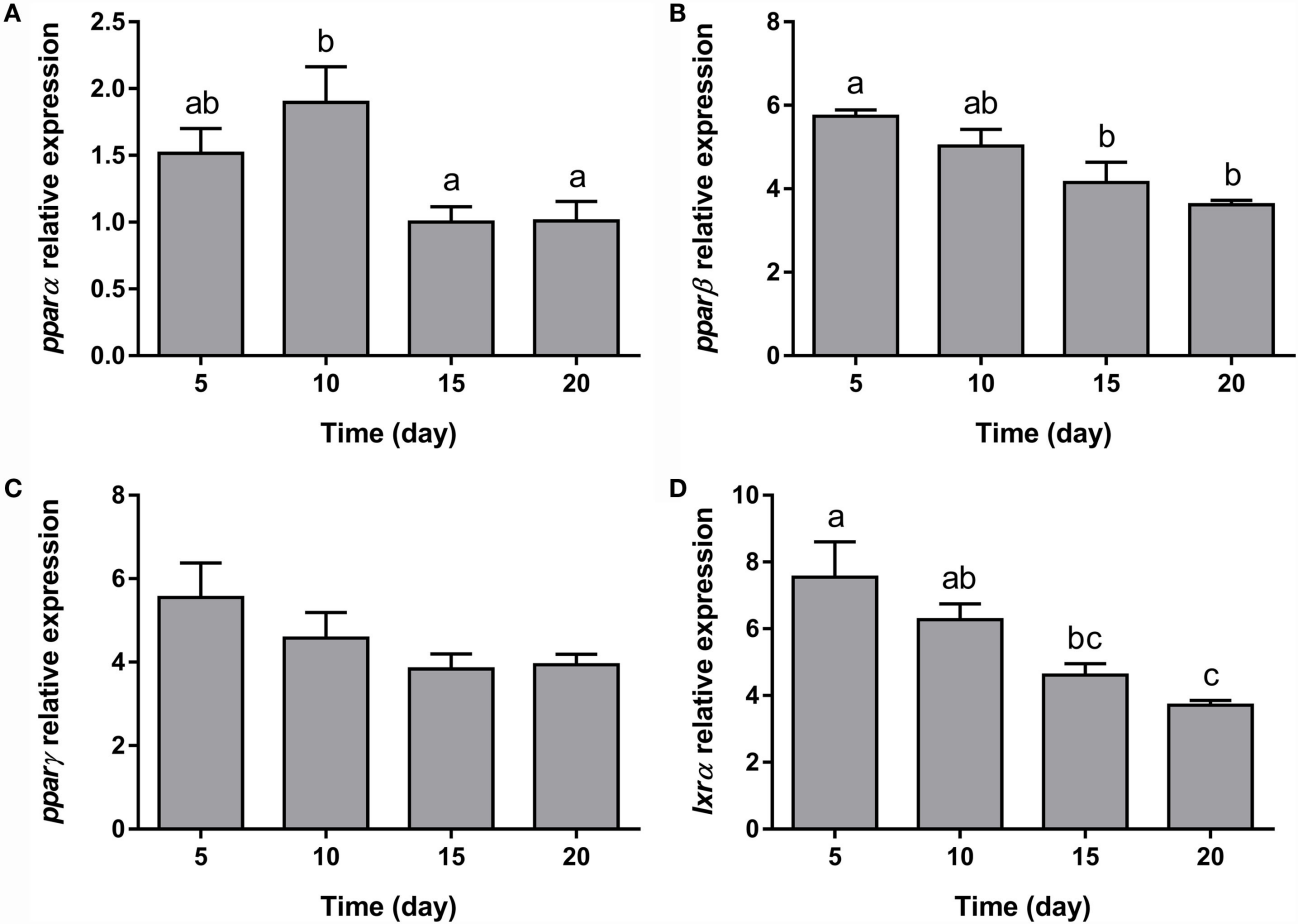
**Gene expression profile of transcription factors in gilthead sea bream bone-derived cells during adipogenesis**. The mRNA levels of *ppar*α **(A)**, *ppar*β **(B)**, *ppar*γ **(C)**, and *lxr*α **(D)** were measured by quantitative real-time PCR and normalized to *ef1*α and *rps18*. Samples were taken from adipocyte-like cells at days 5, 10, 15, and 20 of culture. Values are means ± SEM, *n* = 5–6. Bars with different letters are significantly different (*P* < 0.05). *ppar*α, β, and γ, peroxisome proliferator-activated receptor alpha, beta, and gamma, respectively; *lxr*α, liver X receptor alpha; *ef1*α, elongation factor 1 alpha; *rps18*, ribosomal protein S18.

The expression of all the genes implicated in lipid metabolism analyzed, such as *fas, g6pdh*, and *hsl* (Figures 5B–D), was significantly downregulated during adipogenesis of bone-derived cells. On the other hand, the expression of *lpl, gapdh1*, and *gapdh2* was unaffected (Figures 5A,E,F). Moreover, the gene expression of most of the transcription factors determined in the present study (*ppar*α, *ppar*β, and *lxr*α) was progressively downregulated (Figures 6A,B,D), with the exception of *ppar*γ that continued stable (Figure 6C).

**Figure 7:**
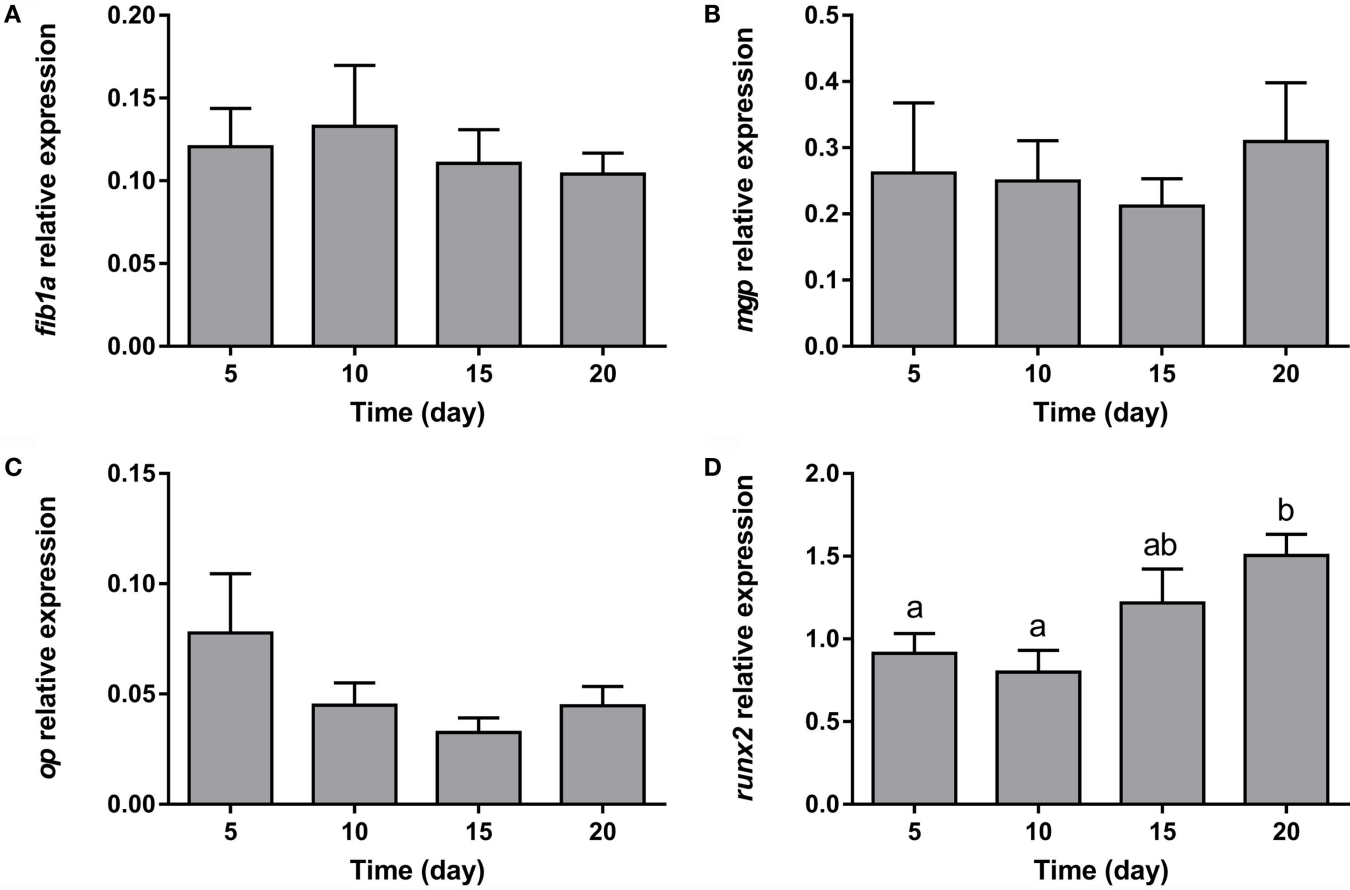
**Gene expression profile of osteogenic markers in gilthead sea bream bone-derived cells during adipogenesis**. The mRNA levels of *fib1a* **(A)**, *mgp* **(B)**, *op* **(C)**, and *runx2* **(D)** were measured by quantitative real-time PCR and normalized to *ef1*α and *rps18*. Samples were taken from adipocyte-like cells at days 5, 10, 15, and 20 of culture. Values are means ± SEM, *n* = 5–6. Bars with different letters are significantly different (*P* < 0.05). *fib1a*, fibronectin 1a; *mgp*, matrix Gla protein; *op*, osteopontin; *runx2*, runt-related transcription factor 2; *ef1*α, elongation factor 1 alpha; *rps18*, ribosomal protein S18.

With regards to the representative osteogenic genes analyzed, the expression of the three components of the extracellular matrix (*fib1a, mgp*, and *op*) remained unaltered during the whole process of adipocyte differentiation (Figures 7A–C). On the other hand, the key transcription factor controlling osteogenesis, *runx2*, showed increasing levels during differentiation (Figure 7D).

## Discussion

Mesenchymal stem cells are multipotent cells that through a two-step process, lineage commitment of specific progenitors and maturation, can be induced to differentiate into cells of several tissue types, such as osteoblasts or adipocytes (20). Local, hormonal, and mechanical factors in MSCs, as well as intermediate precursors and differentiated cells, result in the activation of a series of transcription factors and epigenetic mechanisms, which jointly control the balance of adipogenesis and osteoblastogenesis, including their transdifferentiation (40). Gilthead sea bream MSCs isolated from adipose tissue or vertebra bone can be differentiated into mature adipocytes using a medium supplemented with insulin, IBMX, dexamethasone, and lipid mixture (i.e., DM) (26, 31). Insulin, the most potent of these inducers in mammals, activates *ppar*γ expression (41, 42). IBMX and dexamethasone activate CCAAT enhancer-binding proteins *c/ebp*β and *c/ebp*δ expression, respectively (20), which in turn induce the expression of *c/ebp*α and *ppar*γ, the master regulators of adipogenesis. In preadipocyte primary cultures of some fish species, the addition of lipids in the differentiation cocktail is required to induce full maturation of adipocytes, most probably since polyunsaturated fatty acids, such as docosahexaenoic and eicosapentaenoic acids, are natural activators of Pparγ (43). A lipid mixture containing cholesterol and cod liver oil fatty acids (methyl esters), polyoxyethylenesorbitan monooleate, and d-α-tocopherol acetate has been used in Atlantic salmon (8), rainbow trout (23), large yellow croaker (24), and gilthead sea bream (26). In common carp, linoleic and oleic acids have been employed in combination with T3 and troglitazone (27), whereas in red sea bream (22, 44) and grass carp (25), the use of a DME/F12 medium containing only linoleic acid was enough to induce adipocyte differentiation.

In the present study, the addition of the DM to the preadipocytes downregulated the expression of the early marker *lpl*, as it occurs during the early stages of differentiation in Atlantic salmon preadipocytes (7). However, *lpl* gene expression remained stable during the adipocyte differentiation of bone-derived cells; and in other fish species, such as large yellow croaker (24), rainbow trout (45), and red sea bream (22, 44), its expression was increased after induction of adipocyte differentiation. Thus, the regulation of *lpl* expression could be species-specific, or posttranscriptional mechanisms such as modulation at the activity level cannot be discarded. Fas is an enzyme that regulates the *de novo* biosynthesis of long-chain fatty acids catalyzing the formation of palmitate (46). In this study, *fas* mRNA expression decreased gradually during adipogenesis of both MSC types, contrary to that observed in Atlantic salmon (7) and red sea bream (44) preadipocytes, where its expression was higher and increased during adipocyte differentiation. In a recent study about *de novo* lipogenesis in Atlantic salmon preadipocytes, Bou and coworkers (47) showed that the use of palmitate decreases the expression of acetyl-CoA carboxylase (*acc*), the enzyme that catalyzes the formation of malonyl-CoA necessary for the fatty acid synthesis by Fas. Such data suggested that the synthesis of palmitate mediated by Fas may be blocking its own, and *acc* gene expression, through a negative feedback mechanism, similarly as reported in primary fetal rat calvarial cultured cells where palmitate reduced the expression of *fas* and *ppar*γ (48).

G6pdh, an enzyme of the pentose phosphate pathway that produces the NADPH necessary for the biosynthesis of fatty acids and cholesterol, was upregulated after the induction of differentiation in preadipocytes as it occurs in Atlantic salmon (7), although its expression decreased during adipogenesis of bone-derived cells. In 3T3-L1 cells, Parks and collaborators demonstrated that *g6pdh* overexpression upregulates most adipocyte marker genes (such as *fas* and *ppar*γ) and elevates the levels of cellular free fatty acids and triglycerides. Consistently, *g6pdh* knockdown *via* small interfering RNA attenuated adipocyte differentiation reducing lipid droplet accumulation, indicating that proper expression of *g6pdh* is required for adipogenesis as well as lipogenesis (49). Therefore, the different mRNA expression of *g6pdh*, observed during adipocyte differentiation between the two tissues, suggests that G6pdh may be playing different roles during adipogenesis (i.e., fatty acid synthesis and/or oxidation protection), and thus it can be used as a feature to identify adipocytes derived from each tissue type. Moreover, the lipolytic marker *hsl* decreased transiently its gene expression with differentiation to then increase again, in preadipocytes and to a lesser extent in bone-derived MSCs as well, in agreement with a previous study in human preadipocytes where *hsl* mRNA levels rose during adipocyte differentiation (50). This result suggests that the increase of lipid storage in the cells during adipogenesis may also promote lipolysis through Hsl in order to control its own intracellular levels of lipids. Furthermore, Gapdh produces the triglyceride glycerol required for the triglyceride synthesis being considered a late adipogenic marker. The gene expression of both isoforms of *gapdh* decreased along with adipocyte differentiation in preadipocytes but was unchanged in bone-derived MSCs, in contrast to that occurred at a protein level, where Gapdh appeared to increase from preadipocytes to differentiated adipocytes. In grass carp (25), Atlantic salmon (8), and rainbow trout (23), Gapdh gene expression was not determined, but its activity was also increased with adipocyte differentiation suggesting that perhaps some kind of posttranscriptional, translational, and/or protein regulation processes may be occurring (51).

Regarding the analyses of transcription factors, the expression of all *ppars* decreased early (*ppar*α), steadily (*ppar*γ), or late (*ppar*β) during preadipocyte differentiation and were similarly downregulated during the differentiation of bone-derived cells with the exception of *ppar*γ that remained stable. In previous works with Atlantic salmon, the gene expression of *ppar*α and the short form of *ppar*γ increased during differentiation, while *ppar*γ long was induced during the early phase and decreased at later stages of differentiation (52). Other studies in Atlantic salmon showed that the gene expression of *ppar*α and *ppar*β were unchanged during adipogenesis (53), whereas *ppar*γ was upregulated already in subconfluent cells (7), as observed in our study. In red sea bream, *ppar*α mRNA expression increased up to 7 days after induction and then decreased, while *ppar*β and *ppar*γ remained unaffected (44). In grass carp (25) and in large yellow croaker (24), *ppar*γ transcriptional expression increased gradually during cell differentiation. Here, the presence of the Pparγ protein increased from preadipocytes to early differentiated adipocytes, contrary to that occurred at a transcriptional level, suggesting that control at a posttranscriptional level exists. However, the present data about Pparγ protein expression during differentiation are in agreement with a previous study in rainbow trout preadipocytes where the protein level of Pparγ was higher in mature cells than in proliferating cells, suggesting that Pparγ participates as a transcription factor mostly during the early stages of the adipogenic process (23). Finally, gradual downregulation of *lxr*α mRNA levels was detected during adipocyte differentiation of bone-derived cells, while those were stable in differentiating preadipocytes in disagreement with other studies in 3T3-L1 cells (54) and rainbow trout preadipocytes (55), where its expression increased during differentiation. Lxr induction in the late wave of adipogenesis and its activation inhibits adipocyte conversion, increases glucose uptake, glycogen synthesis, cholesterol synthesis, and fatty acids efflux (56). To sum up, *lxr*α and *g6pdh* during maturation of cells from both tissue types showed a similar pattern of gene expression, suggesting that Lxrα can be involved in the expression of *g6pdh*, regulating the pentose phosphate pathway in gilthead sea bream.

The process of osteogenesis can be divided in three stages, commitment, extracellular matrix production, and mineralization. Fib1a, Op, and Mgp are components of the extracellular matrix. Fib1a is related to the initial state of osteoblast differentiation (57), and in previous studies with gilthead sea bream bone-derived cells, its gene expression increased at day 5 after addition of an osteogenic medium and decreased slowly during differentiation (58). On the other hand, Op and Mgp are key molecules during the phase of calcification of the extracellular matrix (59, 60), and in the gilthead sea bream osteoblast culture, its gene expression increased steadily when mineralization of the tissue started (58). These findings contrast with the results obtained here in the presence of an adipogenic media, where the expression of these osteogenic genes remained low and without variations, indicating a change in the transcriptomic profile of these cells when turning into adipocyte-like cells. Moreover, Runx2 and Osterix are considered key transcription factors required for osteogenic differentiation of MSCs (20). Runx2 promotes cell differentiation into immature osteoblasts inhibiting their commitment to the adipocyte lineage, while Osterix is required for the maturation phase (20). Nevertheless, during the *in vitro* osteogenic development of gilthead sea bream, bone MSCs’ differences in *runx2* expression were not observed (58). Rat adipose-derived stem cells infected with a recombinant adenovirus carrying the *runx2* gene, decreased the gene expression of *lpl* and *ppar*γ and reduced lipid droplet formation (61). In precursor cells isolated from visceral fat of Atlantic salmon differentiating into adipocytes or osteoblasts, Ytteborg and collaborators found that *ppar*γ mRNA was absent in cultures given osteogenic medium, and *runx2* when adipogenic medium was added, suggesting that a similar corepressing mechanism also exists in fish (33). Nevertheless, in our study, the gene expression of *runx2* during adipogenesis was high and increased toward the end, explaining perhaps why the *lpl* and *ppar*γ mRNA levels in bone-derived cells were not modified.

In summary, in the present study, we used primary cultures of MSCs derived from adipose tissue or vertebra bone of gilthead sea bream to investigate the expression profile of lipid metabolism-related genes and transcription factors during adipogenesis. Gilthead sea bream preadipocytes and bone-derived cells were able to differentiate into adipocyte-like cells after addition of a DM. However, the revealed gene expression profile during the process of adipogenesis contradicts some previous findings using similar experimental models in another vertebrate species. The differences between the present and other studies can be related to some kind of species or tissue-specific regulation, differences in the composition of the DM used to induce adipogenesis, or due to the existence of posttranscriptional, translational, and/or protein regulation processes. Future experiments will explore if this cell lineage determination provokes the complete activation of adipocyte functions, and that the cells are not only able to accumulate lipids but also have other key characteristics, such as adipokines production or sensitivity to lipolytic or lipogenic stimuli, as it occurs in mature adipocytes.

## Author Contributions

EC and IN conceived the idea and designed the experiments. CS and NR-H performed the cell cultures and analytical procedures and drafted the manuscript. All authors interpreted the data, revised the manuscript, and approved the final version.

## Conflict of Interest Statement

The authors declare that the research was conducted in the absence of any commercial or financial relationships that could be construed as a potential conflict of interest.
